# Alterations in Saliva and Plasma Cytokine Concentrations During Long-Duration Spaceflight

**DOI:** 10.3389/fimmu.2021.725748

**Published:** 2021-08-24

**Authors:** Stephanie S. Krieger, Sara R. Zwart, Satish Mehta, Honglu Wu, Richard J. Simpson, Scott M. Smith, Brian Crucian

**Affiliations:** ^1^KBR, Human Health and Performance Directorate, Houston, TX, United States; ^2^University of Texas Medical Branch (UTMB), Preventive Medicine and Population Health, Galveston, TX, United States; ^3^JES Tech, Human Health and Performance Directorate, Houston, TX, United States; ^4^National Aeronautics and Space Administration (NASA) Johnson Space Center, Human Health and Performance Directorate, Houston, TX, United States; ^5^Department of Nutritional Sciences, University of Arizona, Tucson, AZ, United States; ^6^Department of Pediatrics, University of Arizona, Tucson, AZ, United States; ^7^The University of Arizona Cancer Center, Tucson, AZ, United States; ^8^Department of Immunobiology, University of Arizona, Tucson, AZ, United States

**Keywords:** cytokine, immune system, spaceflight, saliva, plasma

## Abstract

Long-duration spaceflight is known to cause immune dysregulation in astronauts. Biomarkers of immune system function are needed to determine both the need for and effectiveness of potential immune countermeasures for astronauts. Whereas plasma cytokine concentrations are a well-established biomarker of immune status, salivary cytokine concentrations are emerging as a sensitive indicator of stress and inflammation. For this study, to aid in characterizing immune dysregulation during spaceflight, plasma and saliva cytokines were monitored in astronauts before, during and after long-duration spaceflight onboard the International Space Station. Blood was collected from 13 astronauts at 3 timepoints before, 5 timepoints during and 3 timepoints after spaceflight. Saliva was collected from 6 astronauts at 2 timepoints before spaceflight, 2 timepoints during and 3 timepoints following spaceflight. Samples were analyzed using multiplex array technology. Significant increases in the plasma concentration of IL-3, IL-15, IL-12p40, IFN-α2, and IL-7 were observed during spaceflight compared to before flight baseline. Significant decreases in saliva GM-CSF, IL-12p70, IL-10 and IL-13 were also observed during spaceflight as compared to compared to before flight baseline concentrations. Additionally, plasma TGFβ1 and TGFβ2 concentrations tended to be consistently higher during spaceflight, although these did not reach statistical significance. Overall, the findings confirm an *in-vivo* hormonal dysregulation of immunity, appearing pro-inflammatory and Th1 in nature, persists during long-duration orbital spaceflight. These biomarkers may therefore have utility for monitoring the effectiveness of biomedical countermeasures for astronauts, with potential application in terrestrial research and medicine.

## Introduction

Spaceflight thrusts astronauts into a unique environment characterized by microgravity, circadian misalignment, isolation, confinement, and stress, along with a semi-closed food system, and higher exposure to space radiation. All these factors can cause detrimental effects to the human immune system. Immune dysregulation, including altered leukocyte distribution, alterations in plasma cytokines, reduced T-cell function, and reactivation of latent herpesviruses, persists in astronauts during long-duration orbital space missions (1–3). Clinical events including rashes, hypersensitivity and atopic dermatitis have been reported in astronauts and are potentially related to immune system dysregulation (2, 4, 5).

Cytokines are a broad group of secreted signaling proteins that activate distinct cellular functions and affect various types of immune cells. Chemokines are a subset of small cytokines which act as a chemoattractants to prompt the migration of leukocyte subpopulations and non-hematopoetic cells (6, 7). A previous survey of 22 cytokines in astronauts found significantly increased plasma IL-8, IL-1ra, Tpo, VEGF, and CXCL5/ENA-78 concentrations during flight (8). These data confirm that *in-vivo* hormonal control of immunity is dysregulated during flight. Cytokines, with pleotropic effects that generally preclude their use in specific diagnoses, remain informative in determining immune compromise or general prognosis. For example, Zajkowska et al. found serum concentrations of IL-17, IL-23, IL-21, IL-4 and IL-12 were significantly higher in herpes zoster patients compared to controls (9). Astronauts were found to be shedding VZV DNA in their saliva at levels that overlapped zoster patients (10). Astronaut plasma cytokine profiles are similar to those of zoster patients (10).

Previous research has shown that long-term stress can dysregulate immune response and alter the Th1/Th2 cytokine balance leading to low-grade inflammation (11). Interleukin-6 is a well-known biomarker of inflammation in response to psychosocial stress. Therefore, plasma cytokine concentrations can serve as an indicator of health status and homeostasis. Recent findings have determined that the immune dysregulation is less profound in more International Space Station (ISS) astronauts, as compared to the earlier construction-era crewmembers, implying stress may be a primary factor in astronaut immune dysregulation (12).

Terrestrial clinical findings reveal that saliva cytokines are a sensitive biomarker for both stress and inflammation (13–15). Wang et al. found prominent increases in Th1 and inflammatory cytokines in the saliva of veterans diagnosed with post-traumatic stress disorder (16). Slavish et al. determined that levels of several inflammatory cytokines, including IL-1β, TNF-α, and IL-6, were elevated in saliva in response to acute stress (17). Additionally, students undergoing acute psychosocial stress have an elevated saliva IL-6 concentration (18). Salivary IL-6 concentrations are positively correlated to serum C-reactive protein concentrations, a key measure of inflammation (19). Studies to correlate saliva and plasma cytokine concentrations have yielded mixed results. In normal subjects there appears to be weak correlation, but in stressed individuals with increased concentration correlation improves (20, 21). Saliva cytokines have not been investigated in astronauts to date but represent an appealing area of interest for spaceflight research because of the non-invasive nature of obtaining samples and straightforward processing and storage procedures. During planned missions to the moon and future long-duration voyages to Mars, obtaining viable venous blood samples likely will not be an option due to costs, mass and volume constraints, and most of all, the limited processing and *in situ* analysis capabilities of these first exploration missions.

Understanding the specific nature of immune compromise in astronauts is essential to the development of potential countermeasures (22). We sought to further characterize the immune dysregulation in current ISS astronauts by investigating 13 previously unreported plasma cytokines before, during and after spaceflight. We also assessed salivary cytokines in astronauts in an attempt to validate saliva as a viable non-invasive biosample for astronaut medical monitoring and clinical research.

## Methods

### Subjects

The subject pool of astronauts participating in the venous blood collection consisted of individuals that ranged in age from 38 to 60 years old at the time of launch and spent between 136 and 290 days in space. The astronauts participating in the saliva collection consisted of individuals who spent between 140 to 290 days in space and ranged in age from 49 to 56 at the time of launch. There were 11 males and 2 females participating in the venous blood collection and 5 males and 1 female participating in the saliva collection. Of the subjects participating in the collections, 6 individuals provided both plasma and saliva. The protocol was reviewed and approved by the Institutional Review Board at the NASA Johnson Space Center, Houston, TX. Subjects provided informed consent before data collection.

### Saliva

Saliva was collected from 6 ISS crewmembers daily for 5 consecutive days at 2 timepoints before launch, designated launch minus (L-) 180 days and L-45, 2 timepoints during flight, designated Mid and Late, and 3 timepoints after landing, designated Return plus (R+) 0 days, R+30 and R+90. For both ground and flight, and for diurnal consistency, samples from all subjects were collected just after waking and before eating breakfast. Samples were collected by saturating a synthetic ‘Salivette’ (Salimetrics, State College, PA) and freezing until processing. This includes inflight samples, which were frozen at -96°C, and maintained frozen until returned to Earth and delivered to the laboratory. Upon delivery, and prior to processing, all samples were thawed, aliquoted and frozen at -80°C until batch processing could be completed. Definition testing confirmed that there is no reduction in cytokine concentrations using the Salivette methods, as compared to passive drool samples (unpublished data).

### Saliva Cytokine Analysis

A protease inhibiter (Sigma, St. Louis MO) was added to the samples after thawing. Samples were mixed and then centrifuged at 10,000 × g for 10 minutes. Samples were diluted 1:2 with assay buffer and analyzed using an EMD Millipore MILLIPLEX MAP Human High Sensitivity T Cell Panel Premixed 13-plex multiplex assay according to the manufacturer’s instructions. The 13 cytokines analyzed are: IL-1β, TNF-α, IL-6, IL-8, IL-2, IFN-γ, IL-4, IL-5, IL-10, GM-CSF, IL-7, IL-12 (p40/p70), and IL-13. Samples were analyzed on a Luminex Magpix instrument to determine sample concentrations. Data are presented as pg/mL to show cytokine concentrations present.

### Plasma

Fasting blood samples were collected from 13 crewmembers on relative days L-180, L-45, L-10, FD15, FD30, FD60, FD120, FD180, R+0, and R+30 into EDTA vacutainers. For the assessment of active TGF-β, performed using a separate kit, analysis was only performed on four crewmembers. Samples were centrifuged soon after and then plasma aliquots from the L-180, L-45, R+0, and R+30 timepoints were removed from the vacutainer and stored in a cryovial at -80°C until batch analysis. After centrifugation, EDTA vacutainers from the L-10 and inflight sessions were immediately frozen at -80°C onboard ISS while still in the gel separator vacutainers until they could be returned to Earth. The L-10 designation reflects preflight samples that were frozen in the tube, on the gel separator, until analysis alongside in-flight samples. While these were often scheduled far earlier than L-10, and most often were collected at the same time as the L-45 sample. After samples were returned to Earth, aliquots of plasma were made and refrozen at -80°C until batch cytokine analysis could be completed.

### Plasma Cytokine Analysis

The samples were analyzed in duplicate using an EMD Millipore MILLIPLEX MAP Human Cytokine/Chemokine Magnetic Bead Panel Premixed 30 Plex Multiplex assay according to the manufacturer’s instructions. Of this array, 19 cytokines had been previously published for the ISS astronauts during spaceflight (8). The previously uninvestigated plasma cytokine data for this publication consists of the 11 cytokines as detailed in **Table 1**. Samples were analyzed on a Luminex Magpix instrument to assess the concentrations of 30 cytokines and chemokines. Additionally, plasma was collected from 4 crewmembers and concentrations of active TGF-β1, TGF-β2, and TGF-β3 were investigated using a Millipore Multiplex kit and analyzed on a Luminex MAGPIX instrument.

**Table 1:** Cytokines assessed in blood plasma or saliva of ISS Astronauts.

	Chemokines	Growth Factors	Inflammatory	Anti-cancer
*Plasma*	Eotaxin	EGF	IL-12p40	IFNα2
	IP-10	IL-3	IL-12p70	TNFβ
		IL-7	IL-13	
		IL-15		
		TGFβ1		
		TGFβ2		
*Saliva*		GM-CSF	IL-1β	IFNγ
		IL-7	IL-6	IL-2
			IL-8	IL-4
			IL-12p70	IL-5
			IL-13	IL-10
			TNFα	

### Statistical Analysis

For saliva, an average of data from the 5 consecutive days of collection was generated for each cytokine. Data were analyzed by repeated measures 1-way ANOVA with a *post hoc* Bonferroni t-test where all data were compared against preflight (L-180) data (SigmaPlot 12.0, Systat Software, Inc., San Jose, CA). Some tests had to be log or 1/x transformed to achieve normality and equal variance. The only exception is EGF, where, due to similarity in findings between flight and the final ground collection, in flight data were compared to L-10 baseline. For plasma a similar statistical analysis strategy was employed, with all data compared to the L-180 baseline.

## Results

### Plasma Cytokines

**Figure 1:**
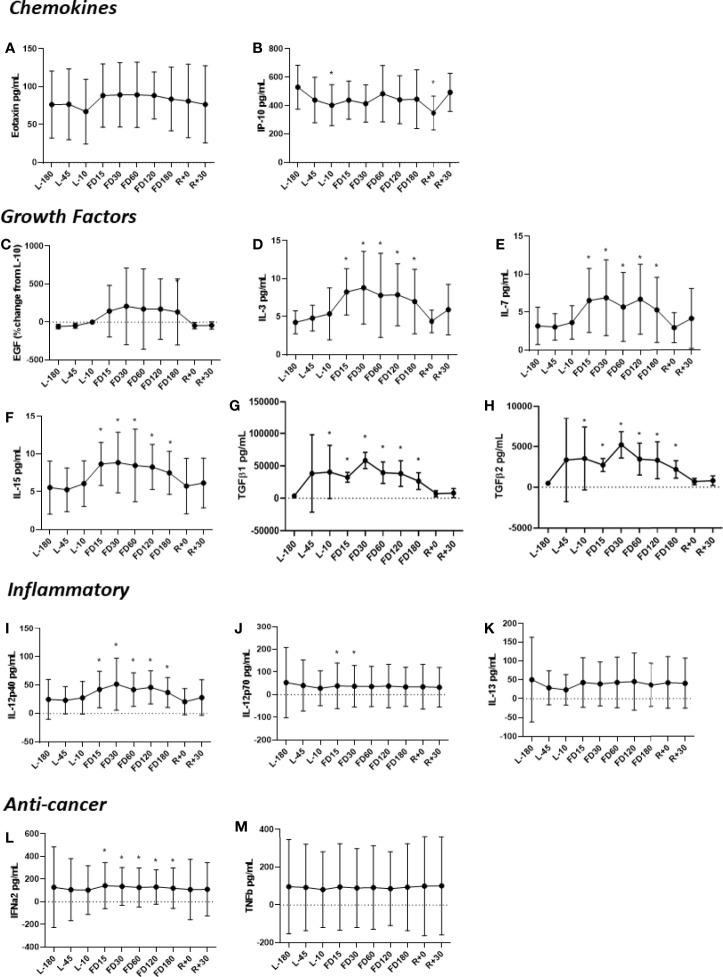
Concentrations of plasma **(A)** Eotaxin, **(B)** IP-10, **(C)** EGF, **(D)** IL-3, **(E)** IL-7, **(F)** IL-15, **(G)** TGFβ1, **(H)** TGFβ2, **(I)** IL-12p40, **(J)** IL-12p70, **(K)** IL-13, **(L)** IFNα2 and **(M)** TNFβ before, during and after spaceflight. The TGFβ assessments **(G, H)** represent quantitation of the active form of the molecule. Data are mean± SD. Significance was evaluated *via* a Student’s t test by comparing all other data points to L-180 baseline data. Significant differences (P ≤ 0.05) are indicated (*).

Concentrations of IL-3, IL-7, IL-15, IL-12p40, TGF-ß1 and TGF-ß2 were all higher during flight compared to the L-180 baseline (**Figure 1**). Concentrations of IL-3, IL-12p40, IL-15, IFNα2 and IL-7 were determined to peak at FD30 with adaption to spaceflight beginning at FD60 and continuing throughout the duration of flight. For all cytokines elevated during flight, concentrations recovered to baseline levels soon after landing. The concentration of IL-12p70 was found to be significantly lower at FD15 and FD30. Eotaxin and EGF were elevated throughout flight until FD180, although not significantly and recovered to near baseline at R+30.

### Saliva Cytokines

**Figure 2:**
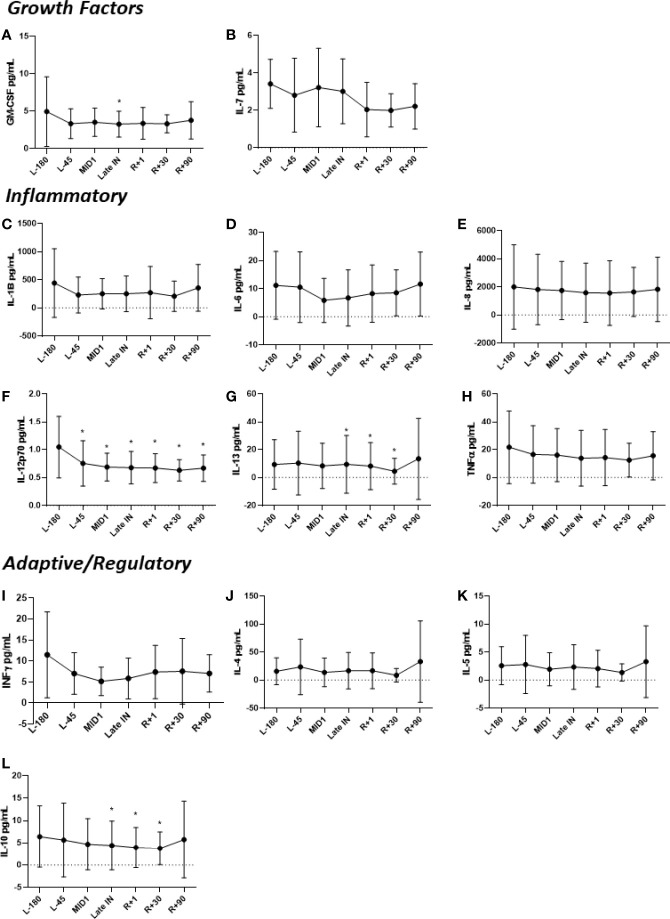
Concentrations of salivary **(A)** GM-CSF, **(B)** IL-7, **(C)** IL-1β, **(D)** IL-6, **(E)** IL-8, **(F)** IL-12p70, **(G)** IL-13, **(H)** TNFα, **(I)** IFNγ, **(J)** IL-4, **(K)** IL-5, and **(L)** IL-10 before, during and after spaceflight. Data are presented in mean± SD. Saliva was collected from crewmembers on 5 consecutive days at the indicated timepoints. Means were determined by grouping preflight, in-flight and postflight data. Significance was evaluated *via* a Student’s t test by comparing all other data points to L-180 baseline data. Significant differences (P ≤ 0.05) are indicated (*).

Salivary cytokines were significantly different during spaceflight, including GM-CSF, IFN-γ, IL-12p70, IL-6 and TNF-α (**Figure 2**). These cytokines were significantly reduced during flight, but returned to preflight levels after landing. For IL-2, all samples had concentrations below the limit of detection, therefore they are not presented.

## Discussion

An earlier assessment of 22 cytokines in astronaut plasma samples found consistent in-flight elevations in cytokines associated with inflammation and other processes (8). This study validated plasma cytokines as a biomarker for *in-vivo* dysregulation of the human immune system during spaceflight. It could not be determined if the causal factor was microgravity, radiation, stress, circadian shifts, altered nutrition, or some synergy therein. Saliva cytokines, an emerging excellent biomarker for stress and inflammation, was not previously assessed in astronauts. The current study assessed the concentration of 13 cytokines in saliva, and 14 previously uninvestigated cytokines in the blood plasma, of astronauts participating in long duration spaceflight aboard the ISS.

Several cytokines were significantly elevated in astronaut plasma during spaceflight as compared to pre-flight samples. These included IL-3, IL-7, IL-15, IL-12p40 and TGF-β. EGF trended towards an increase during spaceflight. We observed a similar trend in cytokine profiles between the 2 sample types, including IL-6, IFNγ, IL-8 and IL-12p70. However, IL-7 and IL-13 did not show any correlation between saliva and plasma. This is not surprising considering Williamson et al. found only IL-6, IFNγ and MIP-1β statistically significantly correlated between passive drool and plasma (23).

Cytokines have diverse roles in regulating immunity. Interleukin-3 (IL-3) is produced by many cell types, including monocytes and macrophages, stroma cells, NK cells, and mast cells but mainly activated T cells (24, 25). It plays an important role in hematopoiesis and induces proliferation and differentiation of myeloid lineage cells (26). IL-15 is secreted after viral infection to induce the proliferation of NK cells to kill virally infected cells (27). IL-15 is expressed by a variety of cell types and tissues, including monocytes, macrophages, fibroblasts, kidney, skeletal muscle, lung, and heart (28). As a pleiotropic cytokine, it plays an important role in innate and adaptive immunity (27). IL-12 has been found to be involved in the differentiation of naive T cells into Th1 cells and is known to play a pivotal role in the activation NK cells and CD4+ T helper lymphocytes (29, 30). Due to its role in the induction of Th1 immune responses, IL-12 has been linked with Th1 mediated autoimmune diseases (31). Interleukin-7 (IL-7) is a hematopoietic growth factor that is critical for lymphocyte survival and development and is required by early T cells and B cells for development in the thymus and bone marrow, respectively (32–34). It is produced by stromal cells in the lymphoid tissues including epithelial cells located in the bone marrow and thymus (35). TGF-β is a family of immunoregulatory cytokines which are secreted by all immune cells lineages, and which possess many distinct functions. TGF-β has been implicated as a regulator for bone formation as well as involved in differential regulation of blood vessel growth in modeled microgravity (36, 37). TGF-β gene expression has been investigated in animals flown in space and has been suggested as a key master regulator in response to spaceflight stress factors (36). It plays a primary role in suppressive and inflammatory immune responses and regulates thymic T cell selection (38). TGF-β is well known to have immunosuppressive functions as well as inhibiting cytotoxic T lymphocytes (CTL) and promoting Th17 cell development (38). Epidermal growth factor (EGF) stimulates proliferation, cell growth and differentiation by binding EGFR. Basal et al. recently found that EGF can promote bone formation and microvascularization in osteonecrosis surgically induced in rats (39).

Collectively, the pattern of novel cytokine alterations described herein, particularly growth factors and cytokines associated with immune mobilization, fits well with previously published findings, adding to a more complete characterization of space flight immune system dysregulation. Increases in IL-3 and IL-15 are consistent with immune mobilization, inflammation or general upregulated *in vivo* responses. Increases in IL-7 may be related to general hematopoietic mobilization. An elevated WBC has been reported to persist during spaceflight (1). The increase in IL-12 is also consistent with immune activation, particularly Th1 and cytotoxic responses. A shift in CTL maturation state has also been previously reported for ISS astronauts (1).

The fact that these cytokines are elevated confirms the previous finding that generically, plasma cytokines represent an excellent biomarker for *in-vivo* dysregulation of immunity in astronauts (8). Conversely, their pleotropic nature precludes their use for specific diagnoses. Changes in clinically relevant biomarkers, such as the reactivation and shedding of VZV DNA, have previously been observed in astronauts (2). Certain adverse clinical events, such as mild infectious diseases or persistent atopic dermatitis, have also been documented in the ISS crewmembers (2, 4, 5). These clinical outcomes remain likely related to the immune dysregulation indicated by the alterations in systemic cytokine concentrations. With an ability to be assessed by multiplex technology on minimal sample volumes, we suggest that plasma cytokines therefore represent an attractive biomarker against which the effectiveness of potential countermeasures may be evaluated (22).

Potentially problematic for deep space missions, assessing plasma cytokine concentrations requires a moderately invasive and time-intensive blood collection. Saliva cytokines therefore may represent an attractive alternative. Saliva is based on a filtrate of plasma and was thought potentially useful as a non-invasive biosample for astronauts. Already, saliva is routinely used for monitoring stress hormones and latent virus reactivation in ISS crewmembers. We performed an assessment of 13 cytokines on crewmember saliva samples collected before, during, and after spaceflight. The plasma and saliva assessments were performed as parts of 2 distinct flight experiments; therefore, the cytokine panels utilized were not the same. The cytokines selected for saliva analysis are represented in **Table 1**. It should be noted that some cytokines may be secreted in saliva at varying concentrations throughout the day, manifesting a diurnal variation. A follow up study may advocate for multiple saliva collections in a single crew day to better address this limitation. However, for this study, operational constraints limited the investigator team to a single collection. To minimize the confounder, samples were consistently collected within 30 minutes after waking. It is known that astronauts lose their circadian entrainment during flight due to the environmental conditions. A future study should advocate for multiple samples, to provide a better assessment of longitudinal alterations in saliva cytokine concentrations, not due to simple diurnal variation.

**Figure 3:**
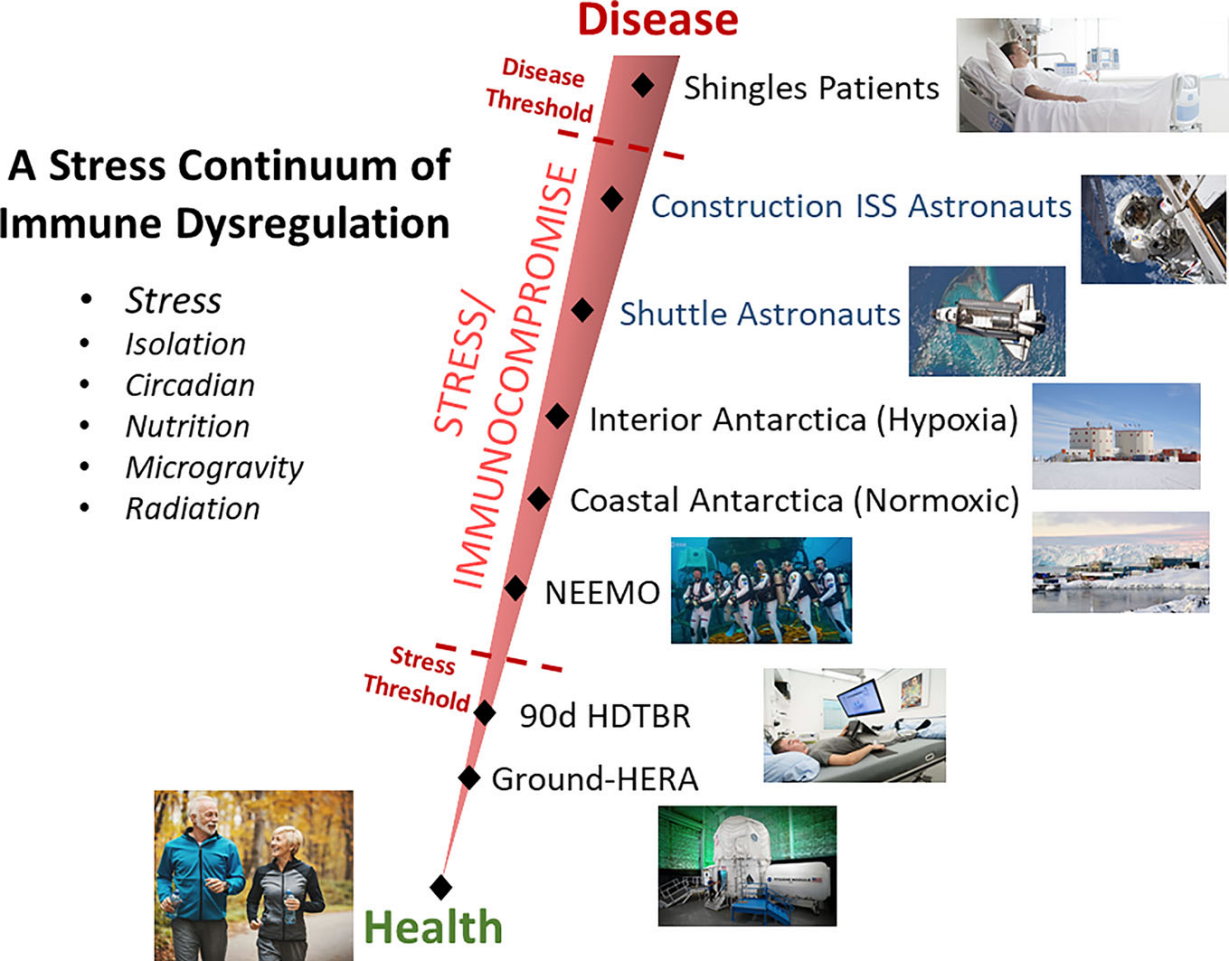
Graphical representation of the proposed relationship between health and stress, detectable using biochemical measurements but with no clinical manifestations, and chronic stress with related adverse clinical events. Stressful conditions, such as spaceflight or various deployment ground analogs of spaceflight, are represented on the continuum based on research findings. Note that current ISS astronauts general experience less stress due to certain deployed biomedical countermeasures (consequently less viral reactivation, improved cortisol levels, etc.), than astronauts during the ‘construction phase’ of ISS and therefore are represented lower on the stress continuum.

Somewhat surprising considering the literature’s supposition that saliva cytokines are a sensitive indicator of stress, was the finding that saliva concentrations of none of the 13 measured cytokines increased during spaceflight. Both GM-CSF and IL-12 p70 were decreased at one or more in-flight timepoints. This is potentially explained by the fact that a >12 year survey of astronaut immunity onboard ISS, from ‘construction era’ to present day ‘science operations phase’ found that more recent crews show improved immunity, reduced inflammation, and reduced concentration of stress hormones (12). This was ascribed to the deployment of several biomedical countermeasures onboard the ISS. It may be that life onboard the ISS is simply ‘less stressful’ than during the construction era and that these biomedical countermeasures were effective. Importantly, that means that stress, and not radiation and microgravity, is a primary causal factor in spaceflight immune dysregulation, and that countermeasures can be deployed to improve immunity in astronauts. We suggest a ‘stress continuum’ exists when considering both measurable stress and clinical disease risk, where construction era astronauts were closer to the ‘disease threshold’ (**Figure 3**). Other ground based ‘analogs’ of spaceflight, such as undersea deployment and Antarctica winterover, may also be placed on this continuum. Quantification of the asymptomatic reactivation of EBV, as part of several distinct investigations (*via* salivary detection of virus DNA), were found to generally increase along the linear increasing order of the analogs as represented on **Figure 3** (40) [unpublished data]. Almost all the described countermeasures deployed to ISS benefiting immunity, including augmented restive and aerobic exercise, more frequent resupply, etc., do not translate to deep space vehicle designs based on size and power limitations. We suggest that continued monitoring during these missions, likely practical given the advent of miniaturized and microfluidics devices, should include plasma or saliva cytokines, to determine the need and effectiveness of deep space countermeasures.

## Data Availability Statement

The raw data supporting the conclusions of this article will be made available by the authors at the NASA Life Sciences Data Archive, upon request and approval.

## Ethics Statement

The studies involving human participants were reviewed and approved by NASA IRB Office of Research Assurance: Research Integrity & Protection of Human Subjects. The patients/participants provided their written informed consent to participate in this study.

## Author Contributions

SK and BC conceptualized and wrote the manuscript with significant inputs from SZ, SS, RS, SM, and HW. Plasma samples were collected on ISS by SZ and SS. BC, SK, RS, SM, and HW are on the investigator team which collected and processed all ISS saliva samples. SK performed ground processing and analysis of all samples. SZ performed statistical analysis. All authors contributed to the article and approved the submitted version.

## Funding

This work was funded via awards to SS and BC by the NASA Human Research Program, Human Health and Countermeasures Element.

## Conflict of Interest

SSK was employed by KBR. SM was employed by JES Tech.

The remaining authors declare that the research was conducted in the absence of any commercial or financial relationships that could be construed as a potential conflict of interest.

## Publisher’s Note

All claims expressed in this article are solely those of the authors and do not necessarily represent those of their affiliated organizations, or those of the publisher, the editors and the reviewers. Any product that may be evaluated in this article, or claim that may be made by its manufacturer, is not guaranteed or endorsed by the publisher.
